# Early changes in spatiotemporal dynamics of remapped circuits and global networks predict functional recovery after stroke in mice

**DOI:** 10.1117/1.NPh.12.S1.S14604

**Published:** 2024-12-20

**Authors:** Ryan M. Bowen, Jake Lee, Brendon Wang, Keith R. Lohse, Hanyang Miao, Jonah A. Padawer-Curry, Asher J. Albertson, Eric C. Landsness, Adam Q. Bauer, Jin-Moo Lee

**Affiliations:** a Washington University in St. Louis, Department of Neurology, St. Louis, Missouri, United States; b Washington University in St. Louis, Department of Biomedical Engineering, St. Louis, Missouri, United States; c Washington University in St. Louis, Department of Physical Therapy, St. Louis, Missouri, United States; d Washington University in St. Louis, Imaging Sciences PhD Program, St. Louis, Missouri, United States; e Washington University in St. Louis, Department of Radiology, St. Louis, Missouri, United States

**Keywords:** stroke, stroke size, stroke recovery, remapping, brain network repair

## Abstract

**Significance:**

Stroke is the leading cause of chronic disability in the United States. How stroke size affects post-stroke repair and recovery is poorly understood.

**Aim:**

We aim to investigate the effects of stroke size on early repair patterns and determine how early changes in neuronal circuits and networks predict functional outcomes after stroke.

**Approach:**

We used wide-field optical imaging, photothrombosis, and the cylinder-rearing assay to examine changes in neuronal circuit and network activity in the context of functional recovery after stroke.

**Results:**

Larger strokes ablating $S1_{\mathrm{FP}}$ caused diffuse and widespread forepaw stimulus-evoked cortical activation, including contralesional regions evolving within 4 weeks post-stroke; smaller strokes resulted in more focused ipsilesional activation. Larger strokes decreased neuronal fidelity and bilateral coherence during stimulation of either the affected or unaffected forepaw within this 4-week period. Mice in the larger lesion group demonstrated hyperconnectivity within the contralesional hemisphere at the resting state. Greater degrees of remapping diffusivity, neuronal fidelity degradation, and hyperconnectivity predicted worse 8-week recovery after statistically controlling for the effect of infarct size.

**Conclusions:**

These results suggest that diffuse patterns of remapping, and desynchronization and hyperconnectivity of cortical networks, evolving early after stroke may reflect maladaptive plasticity, predicting poor long-term functional recovery.

## 1 Introduction

Stroke is one of the leading causes of chronic disability in the United States, affecting more than 795,000 people annually and costing $56.5 billion in direct and indirect costs.^1^ Recovery from stroke is spontaneous, but often incomplete, aided by rehabilitative therapies to regain affected functional ability.^2^^–^^9^ Identifying early predictive neuroimaging biomarkers may not only be helpful for prognostic purposes but may also reveal early brain repair mechanisms that are important for behavioral recovery. Although adaptive patterns of functional brain activity that predict stroke recovery are important, equally important are maladaptive patterns of activity in populations of neurons, which can point to targets for therapeutic intervention. 

Remapping is a term used to describe the migration of function of neuronal circuits damaged by stroke to healthy regions. Remapping of affected function has been explored after stroke recovery, and its correlation with functional outcome is seemingly spatially dependent, with perilesional remapping being strongly associated with improved behavioral recovery^10^^–^^12^ and distant/diffuse remapping being associated with poorer outcomes.^13^^–^^15^ Distant remapping into functionally related cortical regions (as seen in bilateral activation) is generally observed in animal models of large strokes, such as middle cerebral artery occlusion (MCAO).^16^ Similar findings have been noted in human stroke, where functional magnetic resonance imaging has shown bilateral, diffuse task-based activation after stroke.^2^^,^^12^ However, little is known about the threshold of stroke size that results in distant/diffuse remapping. Furthermore, although deficits in the function of limbs ipsilateral to stroke have been documented,^17^^–^^19^ changes that occur in unaffected circuits into which affected functions remap when distant remapping occurs are also poorly understood. Meso- and macroscopic characteristics of remapping that may be predictive of stroke recovery, and whether these characteristics are independent of the initial degree of injury, are yet to be explored.

In addition to remapping, it is likely that the integration of remapped functional circuits into global brain networks is also necessary for favorable recovery. Synchrony of spontaneous neuronal activity across the cortex can be parsed into distinct functional networks, termed resting-state functional connectivity (RSFC) networks, which are consistent within and across subjects.^20^^,^^21^ Normalization of these functional connectivity networks, which are disrupted after focal ischemic stroke,^22^^–^^24^ has also been associated with functional improvement after stroke.^23^^,^^25^^,^^26^ Still, how the extent of injury to a somatotopic domain affects global functional network changes and how these functional network changes may prognosticate recovery after stroke remain unknown. Moreover, the integration of affected remapped circuits into these global functional networks and how changes in affected circuits influence functionally related but uninjured circuits are still poorly understood.

Here, we used a variable-sized photothrombotic stroke model that targets the primary forepaw somatosensory cortex ($S1_{\mathrm{FP}}$) in the left hemisphere to produce infarcts of different sizes in conjunction with wide-field calcium imaging in transgenic Thy1-GCaMP mice (expressing a fusion protein of green fluorescent protein and the calcium-binding calmodulin protein under the Thy1 promoter) to examine the effects of infarct size and degree of injury sustained in $S1_{\mathrm{FP}}$ on remapping, functional network repair, network dynamics, and behavioral recovery. Forepaw somatosensory cortex was used due to its well-characterized nature and an extensive battery of reliable functional assays available. Longitudinal wide-field calcium imaging permitted the monitoring of changes in both local circuits and global brain networks, allowing for the contextualization of multiple scales of repair. Our results indicate that larger infarct sizes cause diffuse, bilateral remapping of affected, and in some cases, unaffected, thalamocortical circuits. Subsequent to diffuse remapping, we observed the development of hyperconnectivity within functional networks of abnormal topography and significant decreases in neuronal fidelity and bilateral coherence during stimulation of both affected and unaffected forepaws after larger photothrombotic stroke. All these measures were independently predictive of worsened functional outcomes after stroke, suggesting that these patterns of remapping and network changes were maladaptive. In contrast, a slightly smaller infarct resulted in the remapping of affected paw function within the original $S1_{\mathrm{FP}}$, no remapping of unaffected paw function, normal functional connectivity topologies, and less severe decreases in neuronal fidelity during affected paw stimulation, all of which predicted better behavioral recovery at week 8.

## 2 Methods

### 2.1 Study Design

**Fig. 1:**
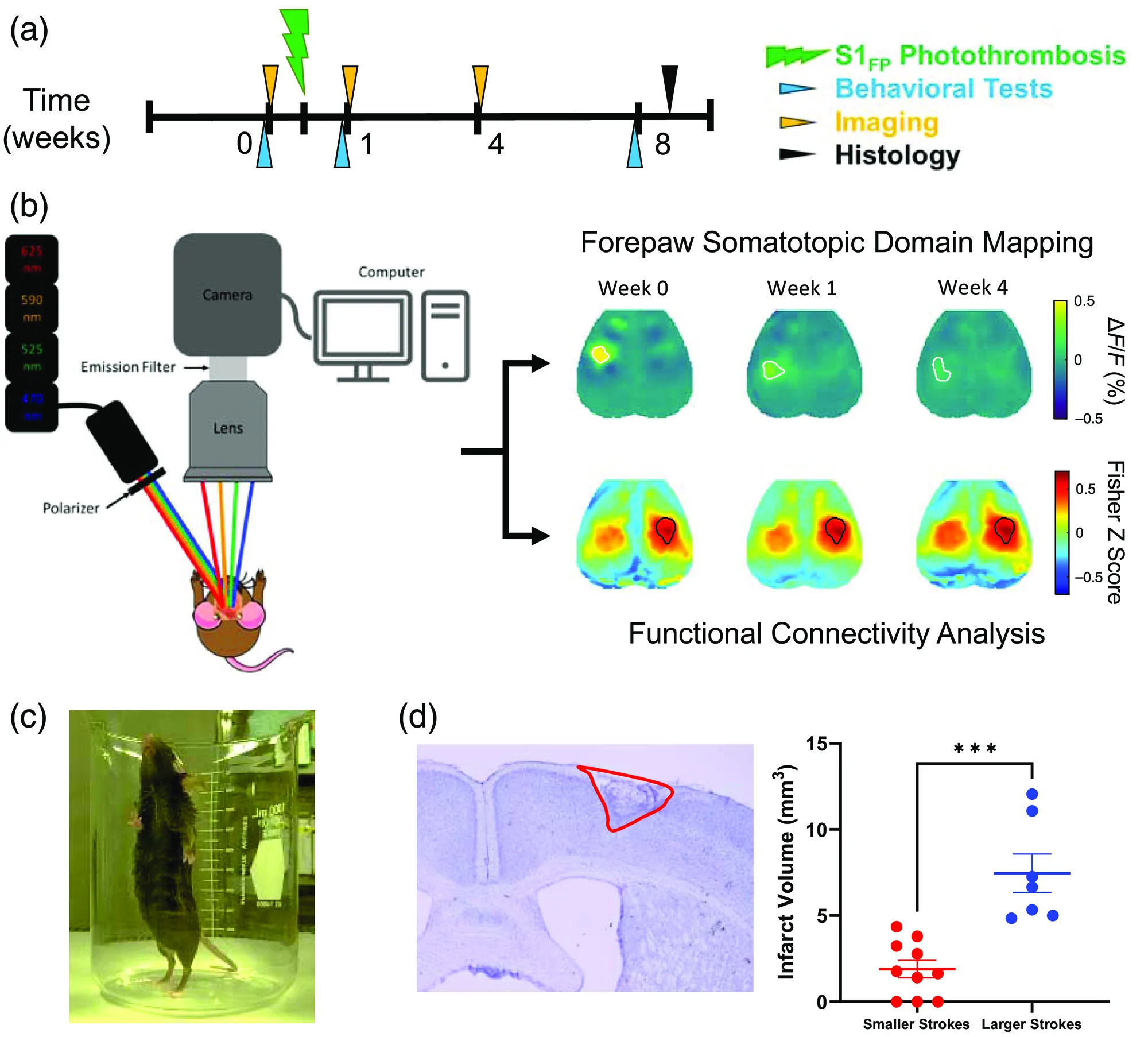
Experimental design to predict long-term stroke recovery using early patterns of repair. (a) A timeline of our experiment is provided showing imaging, behavioral testing, photothrombosis, and histological analysis timepoints. The timeline is given in weeks, where mice were 12 weeks old at time t=0. (b) A diagram of the optical imaging rig used to perform widefield optical imaging on mice (left). Imaging for forepaw somatotopic domain mapping was performed while the mice received anesthetized electrical forepaw stimulation, whereas imaging for functional connectivity analyses was performed on awake, resting-state mice. Example maps of each imaging and analysis paradigm are shown for an individual mouse during each imaging timepoint in the three panels on the right. (c) Somatomotor function was quantified using the cylinder-rearing assay. (d) Infarct volume quantification was performed using Cresyl violet staining; an example image is shown on the left. Histological infarct volumes were significantly larger (p=0.0002) in the larger stroke group (n=7 mice) compared with the smaller stroke group (n=10 mice).

The two primary goals of this study were: (1) to examine the impact of stroke size on remapping, network repair, network dynamics, and behavioral recovery and (2) to investigate multiscale patterns of circuit/network repair that predict behavioral recovery. To accomplish the first goal, mice were administered photothrombotic stroke with a laser diameter focused to either 1 or 2 mm centered on the right forepaw somatosensory cortex (${S1}_{\mathrm{FP}}$). To examine changes in neuronal dynamics potentially predictive of behavioral recovery, mice were only imaged before stroke and 1 and 4 weeks after stroke, whereas behavioral assays were performed before stroke and 1 and 8 weeks after stroke [Fig. 1(a)]. Behavioral assays were performed infrequently to prevent learning effects due to repeated testing.

### 2.2 Mice and Procedures

#### 2.2.1 Ethical approval

All methods below were performed in accordance with Washington University Animal Studies Committee policy in agreement with the American Association for Accreditation of Laboratory Animal Care guidelines.

#### 2.2.2 Mouse model

Mice, raised in enriched housing (as previously described^27^) to increase the dynamic range of recovery following stroke, were given ad libitum food and water with a 12-h–12-h light–dark cycle. The studies described used 12-week-old hemizygous Thy1-GCaMP6f mice on a C57-BL6J background.^28^ Mouse genotype was confirmed using PCR: forward primer 5′-CATCAGTGCAGCAGAGCTTC-3′ and reverse primer 5′-CAGCGTATCCACATAGCGTA-3′.

#### 2.2.3 Cranial windows for optical imaging

Cranial windows were prepared on the dorsal cranium of each mouse as previously described.^29^ Mice were anesthetized with isoflurane (induction 3% to 5%, maintenance 1% to 2%), and head-fixed in a stereotaxic frame. Body temperature was maintained using a feedback-controlled heating pad. The scalp was shaven and sterilized and then incised and retracted along the midline. Metabond dental cement (C&B Metabond, Parkell Inc., Brentwood, New York, United States) was used to adhere a custom Plexiglas window to the mouse’s cranium, completely covering the surgical site. Five days of recovery were allotted before any behavioral or imaging tests were performed.

#### 2.2.4 Photothrombosis

Photothrombosis was performed as previously described.^30^ Mice were administered Rose Bengal via IP injection. A 523-nm diode-pumped solid-state laser illuminated the right forepaw somatosensory cortex (−2.2 bregma, 0.5 lambda) for 10 min. Mice receiving small strokes had laser diameters of 1 mm, whereas mice receiving large strokes had 2-mm laser diameters. Imaging and behavioral tests were not performed for a week following stroke.

#### 2.2.5 Behavioral assays

After stroke to $S1_{\mathrm{FP}}$, mice are known to exhibit asymmetric forepaw use favoring the unaffected limb during the cylinder rearing assay, which then typically become more symmetric as the mouse recovers.^30^^,^^31^ Mice were recorded while performing exploratory rearing behavior for 10 min. The number of frames that each forepaw contacted the wall of the cylinder in the video was recorded and used to calculate forepaw asymmetry using the equation Asymmetry = (Right - Left) / (Right + Left + Both), as previously described [Fig. 1(c)].^30^ Importantly, behavioral recovery was measured as the change in affected (right) forepaw asymmetry from weeks 1 to 8, providing a measure of maximum deficit to the final deficit at 8 weeks (recovery). Recovery was defined as any improvement in asymmetry of the affected paw (greater use of the affected paw) from week 1 to week 8, whereas decline was defined as a decrease in asymmetry of the affected paw (greater use of the unaffected paw).

#### 2.2.6 Infarct volume quantification

Infarct volumes were quantified as previously described.^32^ Mice were deeply anesthetized with pentobarbital and perfused with heparinized phosphate-buffered saline solution. Brains were harvested, frozen on a sliding microtome, and sliced into 40-micron coronal sections. Each successive slice containing infarcted tissue was mounted and stained with cresyl violet. Brightfield images of stained slices were taken using a Keyence BZ-X800 microscope. The cross-sectional area of infarcted tissue on each slice was determined by blind experimenters using ImageJ. Areas were summed and multiplied by the slice thickness to obtain infarct volumes for each mouse [Fig. 1(d)].

### 2.3 Wide-Field Optical Imaging Recordings

#### 2.3.1 Wide-field optical imaging

Cortical calcium and hemoglobin activity were measured using wide-field optical imaging (WFOI), as previously described.^29^ Sequential illumination of the dorsal neocortex was performed using four light-emitting diodes (LEDs) centered on the following wavelengths: 470, 525, 605, and 625 nm. GCaMP was excited with the 470-nm LED, whereas multispectral hemodynamic imaging was performed using the remaining LEDs. Multiwavelength dichroic beam combiners (LCS-BC25-0480, LCS-BC25-0560, LCS-BC25-0585, LCS-BC25-0605, Mightex Systems) were used to collimate and combine all LED sources for illuminating the mouse skull. A cooled, sCMOS camera (Zyla 5.5-USB3, Andor) with a 75-mm f/1.8 camera lens and 80-Hz acquisition framerate (20-Hz acquisition rate for each channel) was used for image detection.

#### 2.3.2 Optical imaging recordings

WFOI was performed as described previously.^32^ Resting state imaging [Fig. 1(b), bottom] for evaluating functional connectivity was performed for 10 min while the mice were awake. Imaging during electrical stimulation of the forepaws [Fig. 1(b), top] was performed following IP injection of a ketamine-xylazine cocktail (100  mg/kg ketamine, 10  mg/kg xylazine). Body temperature was maintained as described above. Forepaw stimulation was performed on each paw for 5 min using computer-controlled, 1-mA electric pulses delivered via microvascular clips (Roboz) attached to either side of the forepaw.^32^ Stimulation was administered in a block design (5-s rest; 5-s of 3 Hz, 1-mA, 0.3-ms pulses; 10-s rest; 15 blocks/5 min recording). Imaging was performed before stroke and 1 and 4 weeks after stroke.

### 2.4 Optical Imaging Processing and Analyses

#### 2.4.1 Optical imaging signal processing

Brain masks were manually created for each mouse, and all subsequent analyses were performed only on pixels labeled as brain. Brain masks and optical imaging sequences were affine transformed to Paxinos atlas space using cranial landmarks.^23^ For all mice and imaging runs, five seconds of dark frames collected were subtracted from all subsequent frames to remove background counts on the sensor. Spatial and temporal detrending were then performed on all brain pixel time traces as previously described.^2^ Oximetric changes were quantified,^33^ and hemoglobin confound was removed from the GCaMP signal, as previously described.^34^ Global signal regression was performed on all brain pixels to expose the topography of functional networks. Imaging runs containing motion artifacts, which were detected by thresholding light levels,^29^ were excluded from analyses.

#### 2.4.2 Evoked response analysis

Regions of interest (ROIs) were defined for forepaw somatosensory cortex by thresholding individual and group-averaged forepaw evoked responses, averaged across blocks and peaks at 50% of the maximum pixel intensity of the peak-averaged response map at each timepoint for each paw. Evoked response maps displayed show group-, block-, and peak-averaged fluorescence signals following delivery of each electrical pulse (0.3 ms at 3 Hz). Individual areas of activation were obtained by counting the number of pixels in individual GCaMP ROIs and converting units to ${\mathrm{mm}}^{2}$ (the length of one side of a pixel was equal to 78 microns). Amplitude-area products were calculated by multiplying an individual mouse’s area of activation by the summed fluorescence contained in that area. Mice with a maximum GCaMP response amplitude of less than 10% of their baseline maximum value were excluded from analyses.

#### 2.4.3 Power and fidelity analyses

Fast Fourier transforms (FFT, native “fft()” function in MATLAB) were performed as previously described^29^ on block-averaged stimulation runs during both affected and unaffected paw stimulations. Topographies of bandlimited power were created by evaluating group-averaged evoked response GCaMP power over 0.1 to 8 Hz. Individual ipsilesional and contralesional fidelity values were calculated as previously described.^29^ Briefly, fidelity maps were created by determining the power of the FFT of each pixel at 3 Hz (the frequency of stimulation) +/−0.1  Hz. Individual mouse fidelity values were obtained by averaging fidelity within individually defined ROIs.

#### 2.4.4 Magnitude-squared coherence analysis

Topographies of evoked response coherence with delivered forepaw stimuli (3Hz) were generated for each mouse by calculating magnitude squared coherence (native “mscohere()” function in MATLAB) between block-averaged activity in the unaffected S1_FP_ (see Sec. 2.4.2) and activity in every other brain pixel during stimulation-on periods. Individual magnitude-squared coherence values were determined by calculating magnitude-squared coherence between an individual mouse’s average activity in the unaffected paw evoked response ROI and affected paw-evoked response ROI at 3 Hz during stimulation-on periods.

#### 2.4.5 Functional connectivity analysis

RSFC analyses were performed as previously described.^29^ Pre-processed resting state data were filtered for a desired frequency range (infraslow: 0.1 to 1 Hz, delta: 1 to 4 Hz, and theta: 4 to 7 Hz). The GCaMP fluorescence time signal within an ROI (also referred to as a seed) was averaged and correlated to the fluorescence time signal of every pixel in the brain space for a given mouse, and Pearson z-correlations were plotted on maps at each week to show longitudinal functional network changes. Individual evoked response ROIs from unaffected paw stimulation at specific time points were used for the calculation of seed-based functional connectivity measures including node degree. Node degree was calculated by counting the number of pixels in the brain space that had a Pearson z-correlation of at least 0.5.

#### 2.4.6 Optical imaging infarct size quantification

Quantification of infarct size in optical imaging recordings was optimized by comparing several functional connectivity and power measures. Homotopy maps were calculated by filtering resting state recordings into one of the desired frequency bands listed in Sec. 2.4.5, and finding Pearson z-correlations of pixels reflected across the midline from one another (mirror image pixels). Pearson z-correlations of homotopy maps calculated 1 week after stroke (week 1) were divided by the analogous Pearson z-correlations of homotopy maps at baseline (week 0) to obtain ratio homotopy maps. Ratio homotopy maps were then binarized by a threshold of 0.2, and the largest contiguous region in the binarized map was taken as the infarct ROI. Ratio-integrated power ROIs were obtained by calculating the ratio of integrated power (0.1 to 8 Hz) in each pixel at week 1 compared with week 0, and thresholding the resulting map for values less than 0.2. Seed-based functional connectivity maps were created as described in Sec. 2.4.5 and ratio seed-based FC maps were calculated in the same ratiometric way homotopy maps were. Different thresholds were used to determine infarct sizes using the ratio seed-based FC maps and are displayed in their respective plots.

### 2.5 Statistical Analyses

#### 2.5.1 Sample size calculation

Mice that died before week 8 of the experiment were excluded from statistical analyses, as were mice with maximum GCaMP fluorescence at week 1 that was less than 10% of their baseline maximum GCaMP fluorescence. With these inclusion criteria, our final sample sizes used in statistical analyses on optical imaging measures were 10 mice in the smaller stroke group, 11 mice in the larger stroke group, and 4 mice in the sham stroke group. Two mice in the larger stroke group died before histology could be performed, and histological slices from two other mice in the larger stroke group were unusable due to staining error. Thus, sample sizes for analyses containing histological infarct volume measurements contained 10 mice from the smaller stroke group and 7 mice from the larger stroke group.

#### 2.5.2 Statistical analysis

All statistical analyses, excluding mediation analyses delineated below, were performed using GraphPad Prism 10.1. Statistical differences in longitudinal optical imaging measures were calculated using two-way repeated measures analysis of variance (ANOVA) tests. Post-hoc Dunnett’s tests for multiple comparisons were performed to compare each group’s mean values at weeks 1 and 4 with its mean pre-stroke value. Linear regressions to predict functional recovery using optical imaging measures were performed using the linear regression analysis function in GraphPad Prism. The threshold for statistical significance was set at p=0.05 for all analyses.

#### 2.5.3 Statistical mediation analysis

Statistical mediation analyses were conducted in R v4.3.0.^35^ Data were visually inspected for univariate and multivariate outliers prior to statistical modeling, none were found, and residuals from the linear models were inspected for normality, homoscedasticity, and influential data points.^36^ Mediation was tested through a series of linear models:^37^^,^^38^

**Model 1**: $Y_{i}∼c(X_{i})+\epsilon_{i}$ (where Y is the outcome of interest)

**Model 2**: $M_{i}∼a(X_{i})+\epsilon_{i}$ (where M is the mediator of interest)

**Model 3**: $Y_{i}∼b(M_{i})+c^{\prime }(X_{i})+\epsilon_{i}$

These models are diagrammed in tripartite figures shown in Figs. S3 and S4 in the Supplementary Material. Across models, the outcome of interest (Y) was always behavioral recovery, defined as the change in affected forepaw asymmetry during exploratory rearing in the cylinder rearing assay from weeks 1 to 8. X and M were different histological or neuroimaging variables (detailed below), but these variables were always collected earlier (from weeks 1 to 4), so all variables temporally precede recovery. Coefficient c reflects the unadjusted relationship between Y and X. Coefficient a reflects the unadjusted relationship between M and X. Coefficient b is the effect of M on Y, controlling for X, and coefficient $c^{\prime }$ is the effect of X on Y controlling for M.

Model 1 is useful for seeing the unadjusted relationship between recovery and a candidate predictor. This path, c, is referred to as the total effect but is not a formal part of the mediation. Instead, the mediation focuses on models 2 and 3 to understand the potential path through the mediator, i.e., X→M→Y, which is referred to as the indirect effect, and any remaining direct effect of X on Y after controlling for M, i.e., X→Y. Statistical significance for the mediation effects was based on the nonparametric bootstrap estimate of the standard error (n=1000 iterations).^38^

## 3 Results

### 3.1 Variance in Photothrombotic Infarct Size

**Fig. 2:**
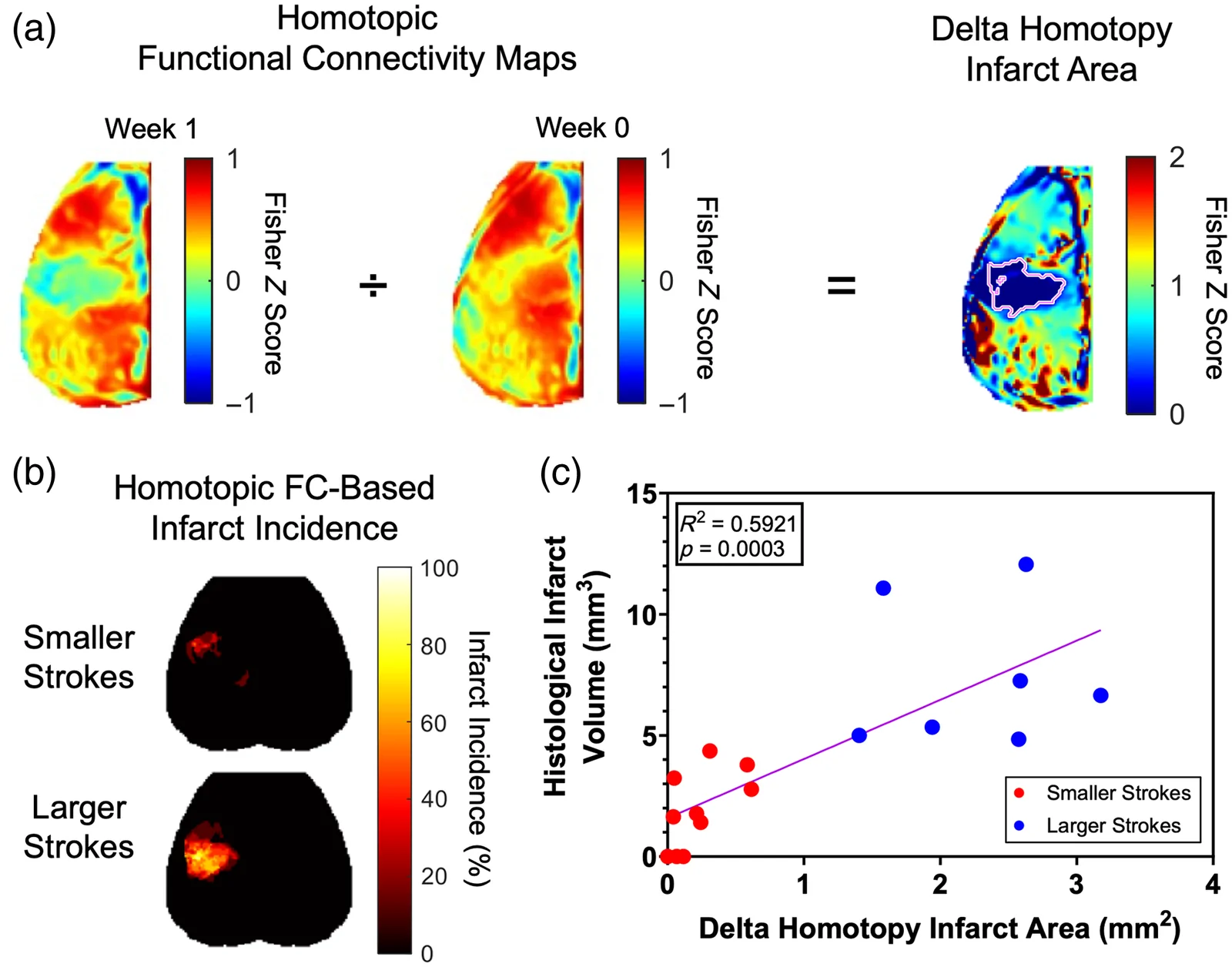
Quantification of infarct size using optical imaging is a reliable proxy of histological infarct volume. (a) Fisher Z scores of week 1 homotopic delta (1 to 4 Hz) FC maps (left) were divided by Z scores of analogous pixels at baseline (middle) to obtain homotopic functional connectivity ratio maps (right). Values 0.2 or lower were classified as delta homotopy infarct areas for each mouse. (b) Incidence maps showing proportions of mice with infarcted tissue across the cortex. (c) Delta homotopy infarct areas at week 1 were plotted against week 8 histological infarct volumes (n=17 mice). $R^{2}$ and p values are displayed on the plot.

Mice were subjected to photothrombotic strokes of two sizes by varying laser diameters (1 versus 2 mm). Infarct size, measured using cresyl violet staining at week 8, was significantly different between the two groups (smaller strokes: $1.899\pm 1.610\;{\mathrm{mm}}^{3}$; larger strokes: $7.462\pm 2.954\;{\mathrm{mm}}^{3}$; p=0.0002) [Fig. 1(d)]. To obtain a reliable imaging proxy for final infarct volume, we tested a 1-week imaging measure: delta-range homotopy maps (functional connectivity maps of mirror-image pixels across the midline), termed delta homotopy infarct maps, as described in the methods [Fig. 2(a)]. Incidence maps for delta homotopy infarcts for each group are shown in Fig. 2(b). Delta homotopy size was significantly correlated to histological infarct volume ($R^{2}=0.5921$, p=0.0003; Fig. 2(c) in those mice that had both measurements. We also tested imaging metrics of theta homotopy infarct maps, integrated power ratio, and contralesional $S1_{\mathrm{FP}}$-seed-based functional connectivity ratio maps in the delta and theta bands [Figs. S1(D)–S1(G) in the Supplementary Material], but none were as strongly correlated to histological lesion volume as delta homotopy infarct maps. In subsequent results, we use delta homotopy infarcts as a proxy for the final infarct volume for regression analyses.

### 3.2 Altering $S1_{\mathrm{FP}}$ Stroke Size Elicits Markedly Different Remapping Patterns

**Fig. 3:**
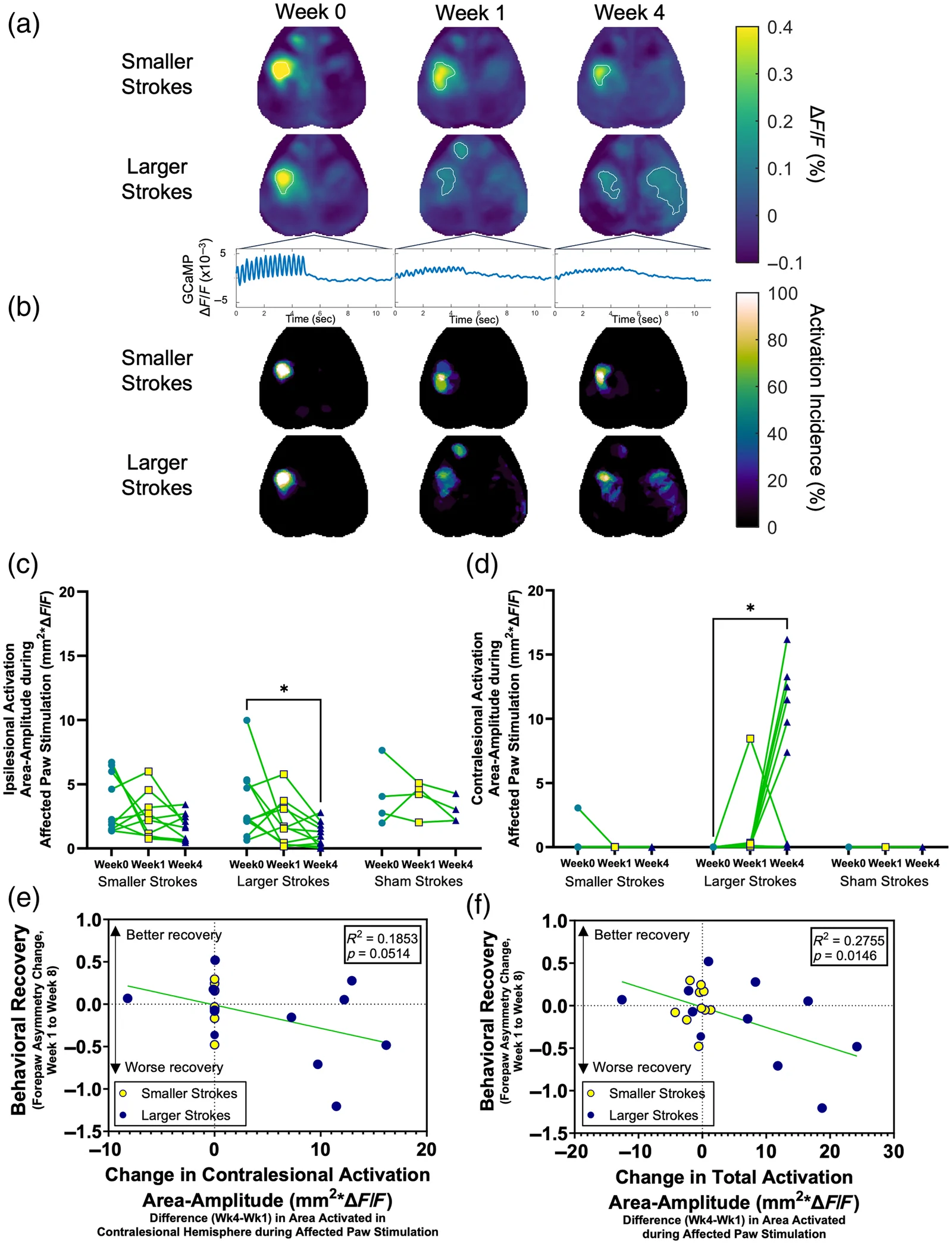
Altering $S1_{\mathrm{FP}}$ stroke size elicits differential changes in early spatial remapping patterns of affected paw circuits. (a) Group-averaged, block-averaged, and peak-averaged GCaMP fluorescence maps during affected forepaw stimulation are shown at each timepoint for each group. Time traces of GCaMP fluorescence activity show stimulation from time t=0 to t=5  s. (b) Incidence maps of individual areas of activation during affected paw stimulation are shown at each timepoint for each group. Areas of activation in the ipsilesional (c) and contralesional (d) hemispheres from panel (b) were multiplied by the summed fluorescence contained in those areas to obtain amplitude area measures of activation for each mouse. Individual amplitude area in each hemisphere is plotted in spaghetti plots for each group (smaller strokes: n=10 mice; larger strokes: n=11 mice). Two-way ANOVA tests with Dunnett’s tests for multiple comparisons were performed on each group and hemisphere to test for significant changes in the amplitude area measure. * denotes p<0.05. (c) Larger strokes: p=0.0209, n=11 mice. (d) Larger strokes: p=0.0146, n=11 mice. (e), (f) Changes in individual amplitude areas of activation from weeks 1 to 4 in the contralesional hemisphere (e) and across the entire cortex (f) are plotted against each mouse’s change in forepaw asymmetry from weeks 1 to 8 (n=21 mice). Mice that received smaller strokes are shown as yellow circles outlined in dark blue; mice that received larger strokes are shown as solid dark blue circles. $R^{2}$ and p values are displayed on each plot.

To examine how infarct size influences remapping and behavioral recovery, we administered photothrombotic stroke of varying sizes centered in $S1_{\mathrm{FP}}$ and performed wide-field calcium imaging during peripheral forepaw stimulation 1 and 4 weeks after stroke [Figs. 1(a) and 1(b)]. GCaMP fluorescence is strongly associated with population-level neuronal activity,^39^ providing a mesoscopic view of cortical neuronal activity. Mice show attenuation of stimulus-evoked GCaMP fluorescence after stroke, leading to diffuse responses [Fig. 3(a)]. Thresholding at consistent values across timepoints therefore poses a problem when using the area of activation during stimulation as a metric of remapping (i.e., areas of activation will be more spatially diffuse and exhibit smaller changes in GCaMP fluorescence after stroke). Therefore, we used the product of the area of activation and the summed fluorescence contained within that area as a metric of remapping, termed “amplitude area.” Representative group-averaged fluorescence maps during stimulation at each time point for each group are shown in Fig. 3(a). One week after stroke, mice that received smaller strokes demonstrated remapping within or near the original $S1_{\mathrm{FP}}$ but did not demonstrate any significant changes in the amplitude areas of their responses [Figs. 3(c)–3(d)]. However, mice with larger strokes were observed to have bilateral and diffuse remapping, with significantly lower amplitude areas of activation in the ipsilesional hemisphere [Fig. 3(c)] and significantly greater amplitude areas of activation in the contralesional hemisphere [Fig. 3(d)] at week 4 compared with week 0. Surprisingly, only a small difference in infarct size resulted in contralesional activation. Mice with infarct sizes greater than or equal to $1\;{\mathrm{mm}}^{2}$ demonstrated contralesional activation, whereas mice with infarct volumes less than or equal to $0.8\;{\mathrm{mm}}^{2}$ did not demonstrate contralesional activation. Indeed, it appeared that a threshold infarct size anywhere from 0.8 to $1.0\;{\mathrm{mm}}^{2}$ (the histological infarct volume equivalent of 4.4 to $4.8\;{\mathrm{mm}}^{3}$) triggered contralesional activation.

To determine if early changes in remapping patterns of affected paw function predicted 8-week behavioral recovery, we correlated early changes in amplitude areas of remapping to changes in forepaw asymmetry between weeks 1 and 8 using the cylinder rearing test. Early changes in contralesional [Fig. 3(e)] and total [summed ipsilesional and contralesional amplitude areas, Fig. 3(f)] stimulus-evoked activation amplitude area (from weeks 1 to 4) predicted forepaw recovery at week 8. Larger amplitude areas of stimulus-evoked activation forecasted worsened behavioral outcomes. Mediation analyses of these data showed that both contralesional and total activation amplitude areas predicted functional recovery, independent of infarct size [contralesional activation: p=0.021 and total activation: p=0.008; Figs. S3 (A) and S3(B) in the Supplementary Material].

**Fig. 4:**
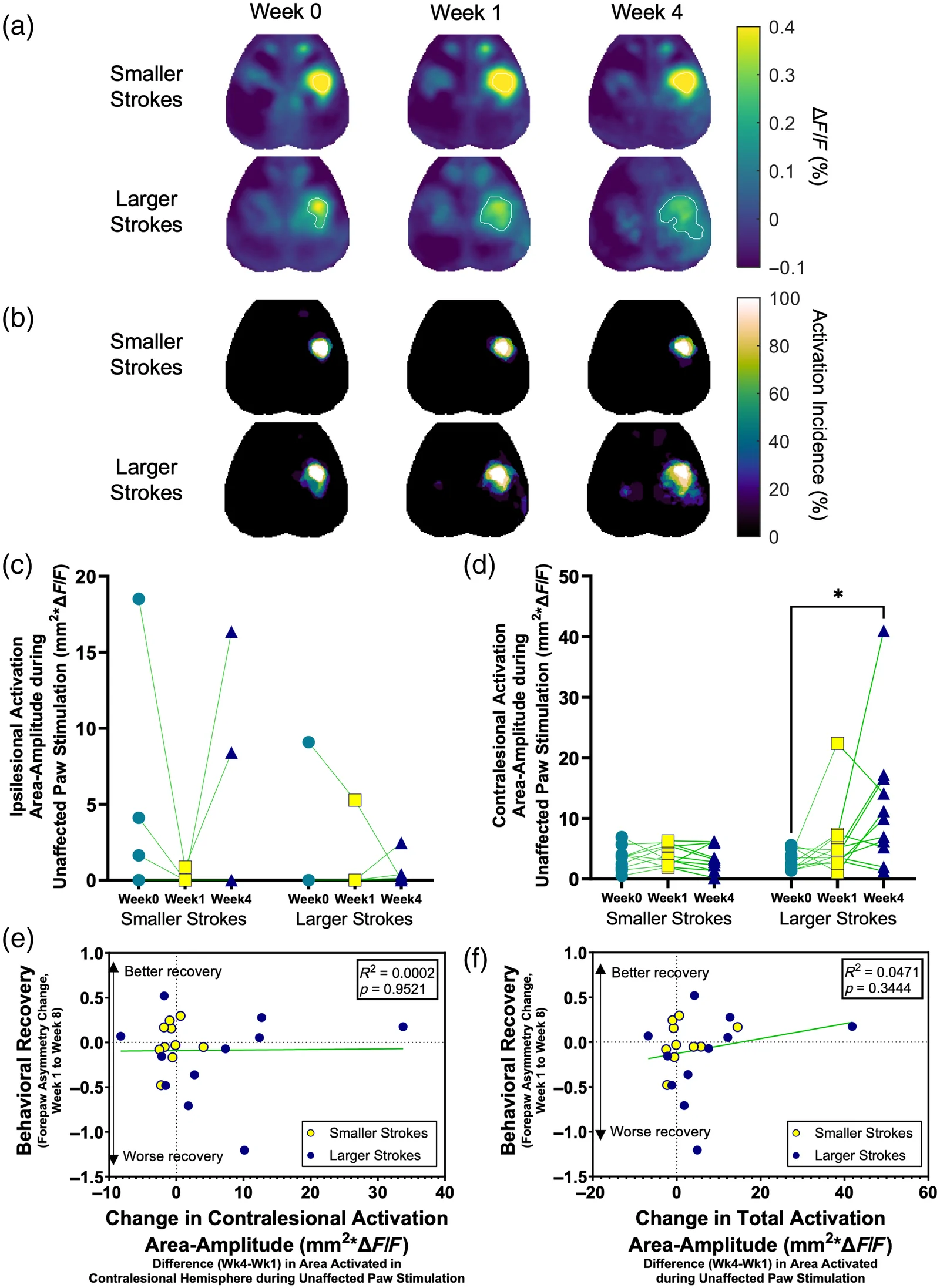
Larger stroke in $S1_{\mathrm{FP}}$ causes diffuse remapping of unaffected forepaw circuits. (a) Group-averaged, block-averaged, and peak-averaged GCaMP fluorescence maps during unaffected forepaw stimulation are shown at each timepoint for each group. (b) Incidence maps of individual areas of activation during unaffected paw stimulation are shown at each timepoint for each group. Areas of activation in the ipsilesional (c) and contralesional (d) hemispheres from (b) were multiplied by the summed fluorescence contained in those areas to obtain amplitude-area measures of activation for each mouse. Individual area amplitude in each hemisphere is plotted in spaghetti plots for each group (smaller strokes: n=10 mice; larger strokes: n=11 mice). Two-way ANOVA tests with Dunnett’s tests for multiple comparisons were performed for each group and hemisphere to test for significant changes in the amplitude area measure. * denotes p<0.05. (d) Larger strokes: p=0.0498, n=11 mice. Changes in individual amplitude area of activation from weeks 1 to 4 in the contralesional hemisphere (e) and across the entire cortex (f) are plotted against each mouse’s change in forepaw asymmetry from weeks 1 to 8 (n=21 mice). Mice that received smaller strokes are shown as yellow circles outlined in dark blue; mice that received larger strokes are shown as solid dark blue circles. $R^{2}$ and p values are displayed on each plot.

Perilesional remapping is typically observed after photothrombotic stroke in mice,^40^^,^^41^ whereas remodeling of distant cortical circuits, such as those in the contralesional hemisphere, typically occurs after large strokes, such as MCAO.^42^ Surprisingly, mice with larger strokes (but still ultimately small in total cortical area) exhibited remapping of unaffected (left) paw function as well (Fig. 4). Larger strokes caused significant increases in unaffected paw evoked-response activation amplitude areas at week 4 in the contralesional hemisphere [Fig. 4(d)], whereas no significant changes in evoked response contralesional activation amplitude areas were observed in mice with smaller strokes during unaffected paw stimulation. There were instances of mice with larger stroke demonstrating ipsilesional evoked response activation during unaffected paw stimulation as well, although changes in ipsilesional activation amplitude areas were not significant in either group [Fig. 4(c)].

### 3.3 Neuronal Fidelity Is Differentially Disrupted after S1_FP_ Stroke and Is Infarct-Size-Dependent

**Fig. 5:**
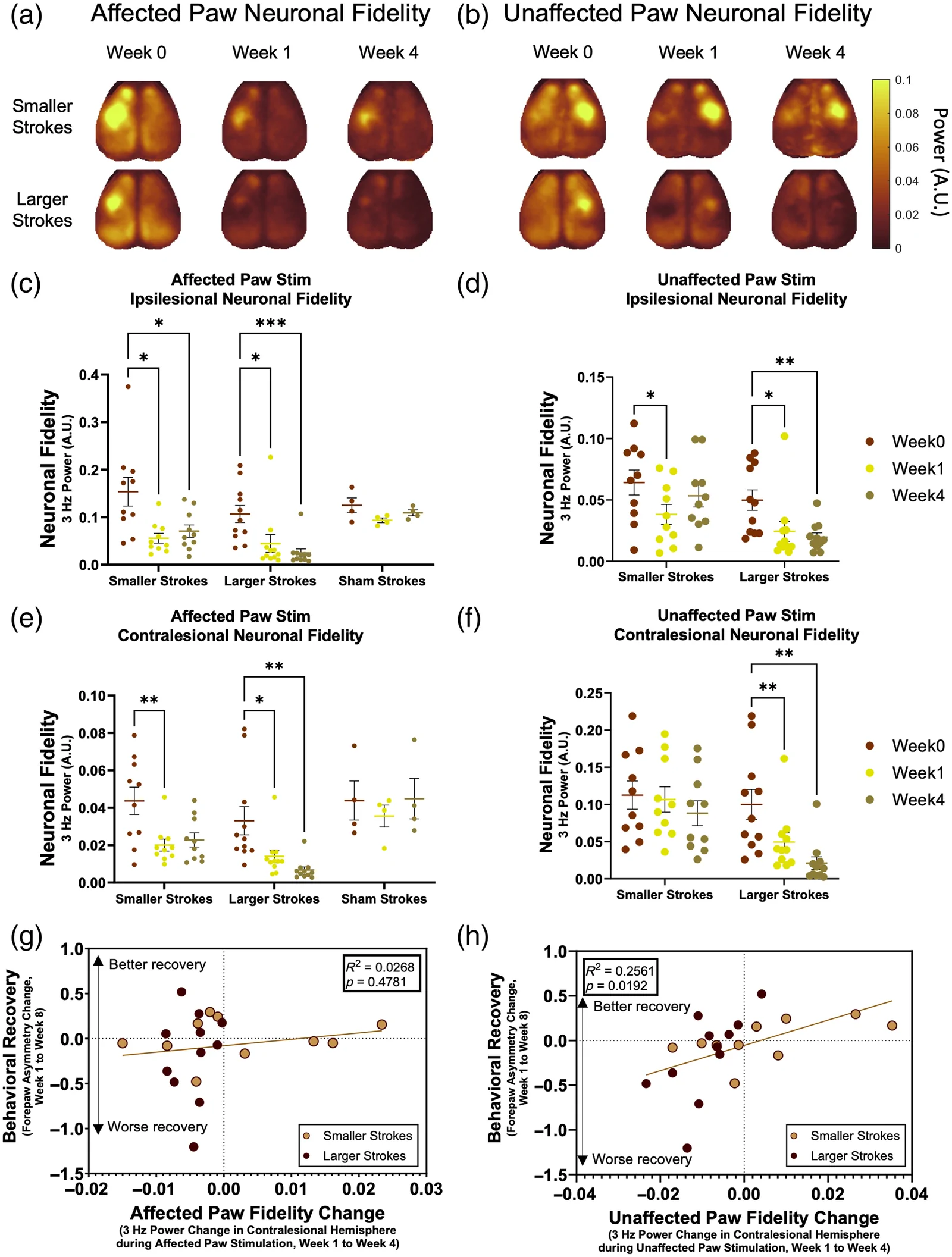
Neuronal fidelity of affected and unaffected circuits is differentially impacted in an infarct size-dependent fashion after $S1_{\mathrm{FP}}$ stroke. Group-averaged, block-averaged 3-Hz power maps denoting neuronal fidelity to stimulation are shown for each group and timepoint during affected (a) and unaffected (b) forepaw stimulation. (c)–(f) Fidelity is quantified in the ipsilesional $S1_{\mathrm{FP}}$ using areas of activation obtained during affected paw stimulation (Fig. 3) and in the contralesional $S1_{\mathrm{FP}}$ using areas of activation obtained during unaffected paw stimulation (Fig. 4). Fidelity is quantified in individual areas of activation in each hemisphere during unilateral stimulation of each forepaw. Individual data points are shown as circles, and means with standard error are shown as horizontal lines with error bars. Two-way ANOVA tests with Dunnett’s tests for multiple comparisons were performed for each group and hemisphere to test for significant changes in fidelity. * denotes p<0.05, ** denotes p<0.01, *** denotes p<0.001. (c) Smaller strokes week 1: p=0.0156, n=10; smaller strokes week 4: p=0.0342, n=10. Larger strokes week 1: p=0.0114, n=11; larger strokes week 4: p=0.0006, n=11. (d) Smaller strokes week 1: p=0.0083, n=10. Larger strokes week 1: p=0.0198, n=11; larger strokes week 4: p=0.0041, n=11. (e) Smaller strokes week 1: p=0.0367, n=10. Larger strokes week 1: p=0.0321, n=11; larger strokes week 4: p=0.0024, n=11. (f) Larger strokes week 1: p=0.0045, n=11; larger strokes week 4: p=0.0012, n=11. Changes in individual neuronal fidelity from weeks 1 to 4 in the contralesional hemisphere during affected (g) and unaffected (h) forepaw stimulation are plotted against each mouse’s change in forepaw asymmetry from weeks 1 to 8 (n=21 mice). Mice that received smaller strokes are shown as light brown circles outlined in dark brown; mice that received larger strokes are shown as solid dark brown circles. $R^{2}$ and p values are displayed on each plot.

In addition to spatial patterns of remapping, it is known that the temporal dynamics of neurons in remapped areas can also influence behavioral recovery.^43^ We investigated changes in temporal dynamics of both ipsilesional and contralesional remapped regions during unilateral stimulation of each forepaw. In particular, we examined neuronal fidelity (the likelihood of a population of neurons to fire in response to a stimulus) over the first 4 weeks after stroke. To quantify fidelity, we measured changes in 3 Hz (the frequency of electrical forepaw stimulation) GCaMP power in both ipsilesional and contralesional regions of evoked activation during stimulation of each forepaw in individual mice (Fig. 5).

As expected during affected paw stimulation, both groups of mice experienced significant attenuations of neuronal fidelity in ipsilesional areas of activation at weeks 1 and 4 compared with week 0, regardless of infarct size [Figs. 5(a) and 5(c)]. Both groups of mice also experienced significant reductions in neuronal fidelity in the contralesional forepaw somatotopic domain during affected paw stimulation at week 1 compared with week 0 [Figs. 5(b) and 5(d)]. Mice that received larger strokes also demonstrated significant decreases in neuronal fidelity in the contralesional forepaw somatotopic domain at week 4 relative to week 0 [Figs. 5(b) and 5(d)].

Unexpectedly, however, the stimulation of the unaffected forepaw differentially induced changes in neuronal fidelity in the contralesional (intact) $S1_{\mathrm{FP}}$ that was dependent on infarct size. Smaller infarcts had no effect on contralesional neuronal fidelity, whereas larger infarcts caused reductions in fidelity at week 1 that worsened at week 4 [Fig. 5(f)]. All mice showed attenuations in fidelity in ipsilesional regions of activation during unaffected paw stimulation at week 1 compared with week 0 [Fig. 5(e)]. Mice with larger strokes demonstrated a sustained reduction in fidelity in ipsilesional remapped regions at week 4 as well [Fig. 5(e)].

Surprisingly, changes in fidelity in the contralesional hemisphere during unaffected paw stimulation from weeks 1 to 4 predicted 8-week affected paw recovery ($R^{2}=0.2561$, p=0.0192)), with larger decreases in unaffected paw fidelity predicting worsening behavioral recovery [Fig. 5(h)]. Changes in fidelity in ipsilesional and contralesional areas of activation during affected paw stimulation [$R^{2}=0.0268$, p=0.4781; Fig. 5(g)], as well as changes in fidelity in ipsilesional areas of activation during unaffected paw stimulation, did not predict functional recovery. Overall, changes in fidelity were stroke-size dependent, and contralesional fidelity changes predicted stroke recovery.

### 3.4 Disruptions in Evoked Coherence of Bilateral Forepaw Somatosensory Cortices Predict Functional Outcome after Stroke

**Fig. 6:**
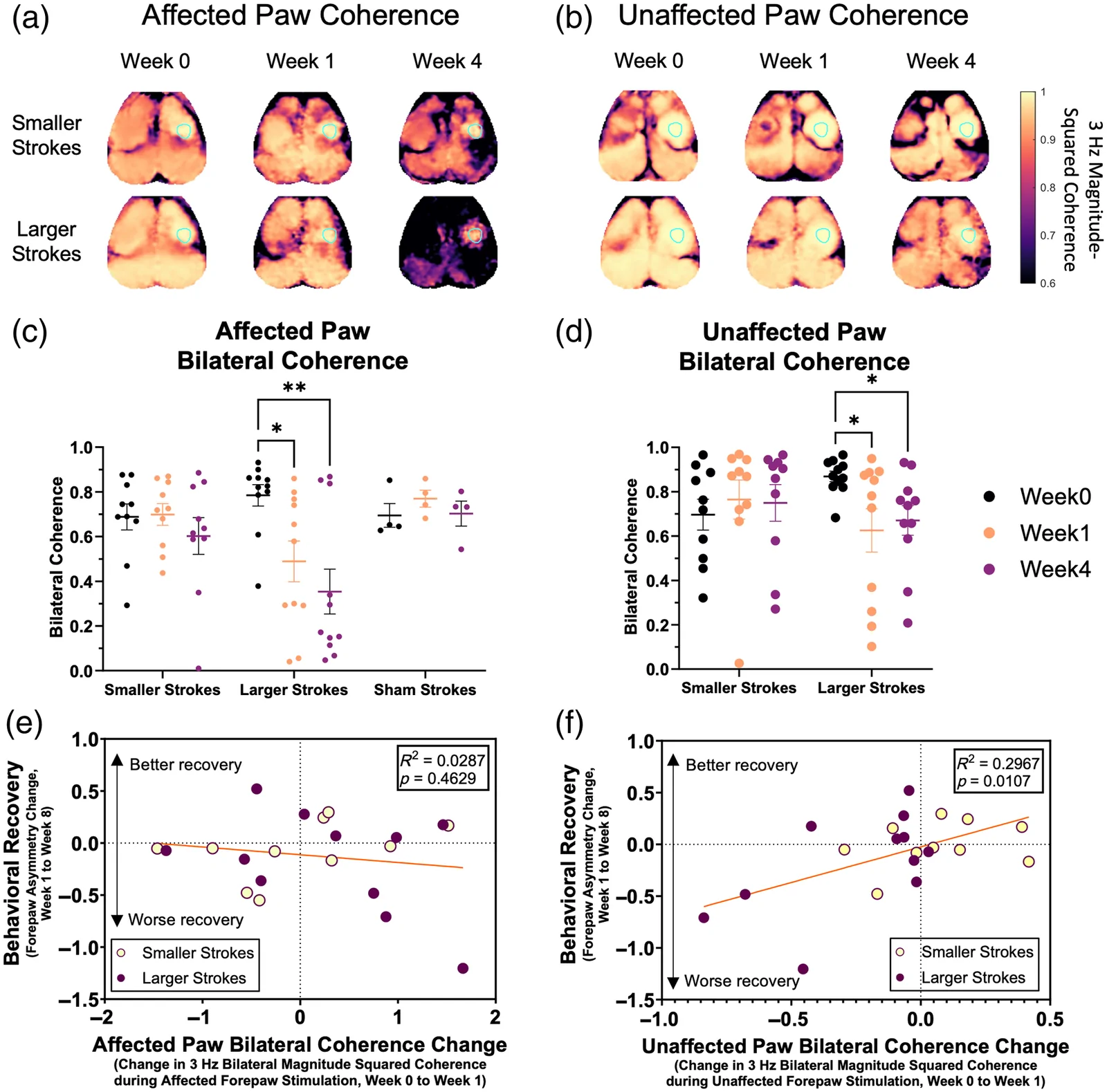
$S1_{\mathrm{FP}}$ stroke size disparately influences bilateral coherence changes during unilateral stimulation of each forepaw. Group-averaged, block-averaged 3-Hz mean-squared coherence maps are shown for each group and timepoint during affected (a) and unaffected (b) forepaw stimulation. ROIs of group-averaged activation during unaffected paw stimulation are outlined in light blue to show approximate locations of average activity used to calculate maps. The quantification of bilateral coherence between individual contralesional $S1_{\mathrm{FP}}$ and ipsilesional $S1_{\mathrm{FP}}$ is displayed during affected (c) and unaffected (d) forepaw stimulation. Individual data points are shown as circles, and means with standard error are shown as horizontal lines with error bars. Two-way ANOVA tests with Dunnett’s test for multiple comparisons were performed for each group and hemisphere to test for significant changes in coherence. * denotes p<0.05, ** denotes p<0.01. (c) Larger strokes week 1: p=0.0135, n=11; larger strokes week 4: p=0.0030, n=11. (d) Larger strokes week 1: p=0.0429, n=11; larger strokes week 4: p=0.0322, n=11. Changes in individual bilateral coherence from weeks 1 to 4 during affected (e) and unaffected (f) forepaw stimulation are plotted against each mouse’s change in forepaw asymmetry from weeks 1 to 8 (n=21 mice). Mice that received smaller strokes are shown as light-yellow circles outlined in purple; mice that received larger strokes are shown as solid purple circles. $R^{2}$ and p values are displayed for each plot.

Our observations of remapping and changes in neuronal fidelity to forepaw stimulation suggest that stroke caused changes in both spatial and temporal patterns of neural activation. We hypothesized that these dynamic changes in thalamocortical circuitry would propagate into affected cortical circuitry and disrupt neuronal fidelity and synchrony of global corticocortical networks. To examine changes in temporal characteristics of global somatosensory networks, we measured 3-Hz mean-squared coherence between contralesional and ipsilesional areas of activation (bilateral stimulation-driven coherence) during individual stimulation of each forepaw (Fig. 6).

Mice with larger strokes had significant decreases in bilateral coherence during unilateral affected paw stimulation 1 and 4 weeks after stroke [Figs. 6(a) and 6(c)], and during unaffected paw stimulation 1 and 4 weeks after stroke [Figs. 6(b) and 6(d)], while no other significant group-level coherence changes were noted for mice having smaller strokes during stimulation of either paw [Figs. 6(a)–6(d)]. Changes in bilateral stimulation-driven coherence during unaffected forepaw stimulation from baseline to week 1 predicted functional outcome [Fig. 6(f)], but changes in coherence during affected paw stimulation did not correlate with or predict behavioral recovery [Fig. 6(e)]. Bilateral coherence changes during unaffected paw stimulation from baseline to week 1 predicted functional outcome independent of infarct size measured by delta homotopy changes, as shown by mediation analysis [Fig. S3(C) in the Supplementary Material].

### 3.5 Larger S1_FP_ Strokes Trigger Hyperconnectivity Within the Contralesional Hemisphere and With Ipsilesional Regions of Remapping

**Fig. 7:**
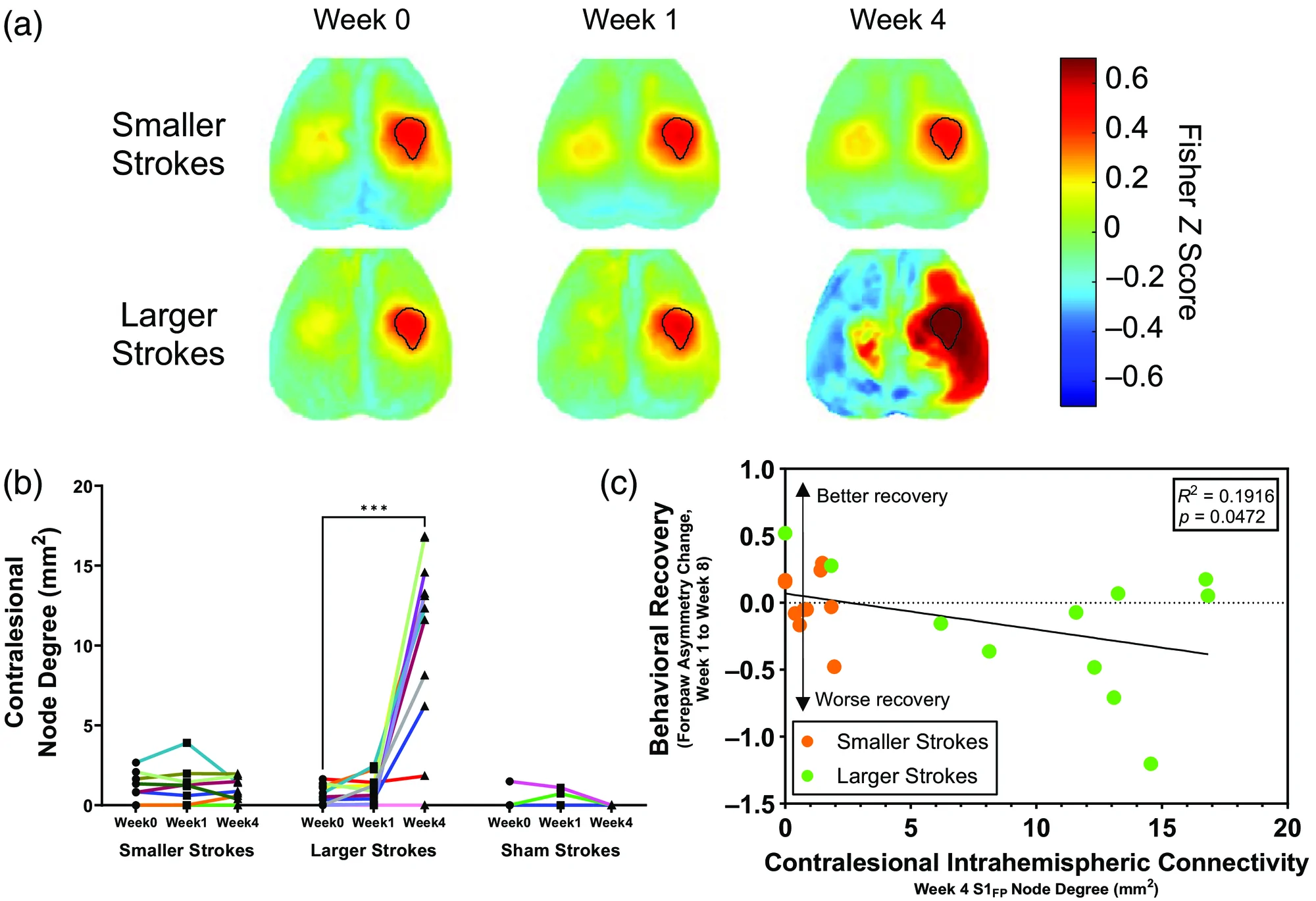
Larger stroke in $S1_{\mathrm{FP}}$ causes substantial and maladaptive hyperconnectivity within the contralesional hemisphere. (a) Group-averaged, delta range (1 to 4 Hz) seed-based functional connectivity (FC) maps are displayed for each group at each timepoint. Maps were obtained by correlating average activity in the ROI outlined in black with activity in every other pixel in the cortex. (b) Node degree of the contralesional hemisphere was quantified by determining the number of pixels in the contralesional hemisphere of an individual mouse’s seed-based FC map with Fisher Z scores of at least 0.5. A two-way ANOVA test with Dunnett’s tests for multiple comparisons was performed for each group to test for significant changes in node degree. Mice with larger strokes had significantly higher contralesional node degrees at week 4 than at week 0 (p=0.0003, n=11). (c) Individual week 4 contralesional node degree values are plotted against each mouse’s change in forepaw asymmetry from weeks 1 to 8 (n=21 mice). Mice that received smaller strokes are shown in orange; mice that received larger strokes are shown in green. $R^{2}$ and p values are displayed on the plot.

To characterize global brain network changes after stroke, we performed awake, resting-state imaging on mice at all timepoints for functional connectivity (FC) analysis. Representative FC maps are shown in Fig. 7(a). We observed that larger strokes of $S1_{\mathrm{FP}}$ caused hyperconnectivity (enhanced FC) between contralesional $S1_{\mathrm{FP}}$ and large portions of the contralesional hemisphere, as well as with ipsilesional remapped $S1_{\mathrm{FP}}$ regions, 4 weeks after stroke. Hyperconnectivity was not observed in mice having smaller strokes.

Changes in spatial patterns of functional connectivity were quantified using node degree, a measure of a region’s degree of connectivity with the rest of the cortex, of contralesional $S1_{\mathrm{FP}}$ cortex with the rest of the contralesional hemisphere [Fig. 7(b)], which showed significant differences between mice that received smaller versus larger strokes. Contralesional node degree 4 weeks after stroke was predictive of behavioral recovery as measured by the cylinder rearing assay, with a higher degree of contralesional hyperconnectivity forecasting worsening functional outcome [Fig. 7(c)]. Mediation analysis showed that week 4 contralesional node degree predicted behavioral recovery independent of delta homotopy infarct size [Fig. S3(D) in the Supplementary Material].

## 4 Discussion

Infarct volume is known to be a salient predictor of recovery after stroke in animal models,^44^ as well as in human stroke.^45^^,^^46^ In this study, we used smaller and larger infarcts centered on $S1_{\mathrm{FP}}$ in mice to examine early changes in functional imaging parameters (reflective of circuit and network plasticity) that predict longer-term behavioral recovery. We found that larger strokes in $S1_{\mathrm{FP}}$ caused diffuse, bilateral remapping of affected paw function with larger activation areas, whereas smaller, partial infarctions of $S1_{\mathrm{FP}}$ elicited focal remapping within the original $S1_{\mathrm{FP}}$. Neuronal fidelity was differentially impacted by stroke size: all strokes unsurprisingly elicited significant attenuations in fidelity during affected paw stimulation, but larger strokes caused markedly greater disruptions in fidelity during unaffected paw stimulation. Larger strokes of $S1_{\mathrm{FP}}$ also induced significant decreases in bilateral coherence during stimulation of either paw, as well as hyperconnectivity in the contralesional, intact hemisphere, findings that were absent in mice that received smaller strokes in $S1_{\mathrm{FP}}$. These results suggest two distinct modes of repair: (1) diffuse remapping of neuronal circuits and hyperconnectivity that predict poor functional recovery and suggest maladaptive plasticity and (2) focal remapping of neuronal circuits and normalized connectivity that predict good functional recovery and suggest adaptive plasticity. These results also suggest that spatiotemporal characteristics of early circuit and network repair influence behavioral recovery in mice and that these properties of plasticity can facilitate the prediction of functional outcomes better than infarct size alone.

### 4.1 Disparate Spatial Patterns of Neuronal Remapping after S1_FP_ Stroke

Remapping as imaged from the cortical surface likely represents repair of damaged excitatory^47^ and inhibitory^48^ cortical circuits, as well as thalamocortical^49^ projections in smaller somatosensory cortical strokes. In our study, smaller $S1_{\mathrm{FP}}$ strokes induced remapping within and nearby $S1_{\mathrm{FP}}$, likely representing the repair of thalamocortical connections. Consistent with this hypothesis, the reorganization of thalamocortical projections to the sensory cortex likely causes mesoscale remapping after small photothrombotic stroke in rats.^50^ Furthermore, optogenetic stimulation of thalamocortical projections enhances motor outcome after small photothrombotic stroke in $S1_{\mathrm{FP}}$ of mice,^49^ suggesting that remodeling of thalamocortical projections heralds good recovery of function. Our data support this finding, demonstrating that mice exhibiting peri-infarct remapping within $S1_{\mathrm{FP}}$, likely due to thalamocortical circuit repair, experienced improved behavioral recovery.

Mice subjected to slightly larger $S1_{\mathrm{FP}}$ strokes exhibited a drastically different remapping pattern. These animals demonstrated diffuse, bilateral activations during affected paw stimulation, exhibiting remapping into ipsilesional regions outside the original $S1_{\mathrm{FP}}$ and into large portions of the spared, contralesional hemisphere. These patterns developed over the first 4 weeks after stroke. Prior work has demonstrated that diffuse, bilateral patterns of remapping are typically observed in stroke patients with large infarcts—such as those with large vessel occlusion (LVO),^51^ and herald poor outcome.^2^^,^^12^ In rodents, MCAO (the mouse equivalent of an LVO stroke in humans) produces a similar diffuse pattern of remapping^16^^,^^42^ and causes more severe behavioral deficits than those produced by smaller photothrombotic lesions.^52^ Our results show similar findings; however, the surprising observation is that a small increase in infarct size can have a major impact on patterns of remapping and recovery. We found that a threshold surface area of 0.8 to $1.0\;{\mathrm{mm}}^{2}$ (the histological infarct volume equivalent of 4.4 to $4.8\;{\mathrm{mm}}^{3}$) resulted in a dramatic transition to diffuse, bilateral remapping patterns. Interestingly, these mice are also those that appear to have complete ablation of the $S1_{\mathrm{FP}}$ based on activation patterns at week 1 (though this measure is not quantified), suggesting the importance of residual healthy tissue in the injured somatotopic domain for robust repair and recovery. Furthermore, the diffuse and bilateral pattern of activation predicted poor recovery at 8 weeks independent of infarct size, suggesting that these circuit changes represented “maladaptive plasticity,” whereas the focal ipsilesional remapping pattern seen in smaller strokes at 4 weeks predicted good recovery at 8 weeks, suggesting “adaptive plasticity.”

Similar contralesional activation was shown just 30 min after stroke in the cortex by Mohajerani et al.^53^ It is important to note that Mohajerani et al. examined neuronal activity in the hyperacute stage, whereas our study examines the chronic stage of stroke recovery. The group also suggests that its results can be explained by rapid synaptic scaling and homeostatic plasticity compensating for loss of function due to stroke.^53^ The results we show happen on the timescale of weeks and, as it is still unclear whether new or existing connections are responsible for aberrant patterns of evoked-response activity, they likely reflect both synaptic scaling and structural changes of stroke-affected circuits. Our results build on the findings of Mohajerani et al., demonstrating that early unmasking causing diffuse and bilateral activations minutes after stroke is maladaptive when those pathways are reinforced, whether that reinforcement is by synaptic potentiation and/or structural remodeling. Given the similarities in evoked response topographies that we and Mohajerani et al. observed at very different timescales, we agree that synaptic scaling of stroke affected circuits should be explored more thoroughly as a potential mechanism of post-stroke repair.

Spatial remodeling of intact circuits not only occurred in affected paw circuits but also in unaffected paw circuits. Our results show significantly larger activation areas during unaffected paw stimulation than at baseline in mice with larger strokes, a trend that was not observed in mice with smaller strokes. The functional significance of this finding is unclear because our behavioral tests assess relative function (right versus left function) but not the absolute ability of individual limbs. However, it is interesting that previous studies reported deficits in unaffected limb function after stroke in humans.^18^^,^^19^ Remapping of unaffected paw function could be due to varying degrees of disinhibition of the contralesional hemisphere caused by varying degrees of ischemic injury.^54^^–^^56^ Greater areas of unilateral cortical injury may cause disinhibition to larger areas of the contralesional hemisphere. This disinhibition results in hyperexcitability in the contralesional hemisphere^57^ and likely mediates changes in temporal dynamics of connected regions in the contralesional hemisphere. Similar to the phenotype of contralesional remapping of affected forepaw circuits, we speculate that the complete ablation of $S1_{\mathrm{FP}}$ is responsible for the remapping of unaffected circuits; the reason why complete ablation of $S1_{\mathrm{FP}}$ seems to be the threshold for these maladaptive patterns of repair remains elusive and should be explored further.

The enhanced activation of the contralesional hemisphere during affected paw stimulation was also progressive: the contralesional areas activated in week 4 were generally larger than those observed in week 1. This growth of the contralesional activation area was strongly predictive of poor behavioral recovery as well, suggesting that this progression was a maladaptive change to brain circuitry. This finding is validated in other studies, which consistently show that the normalization and return of lateralization of evoked responses after stroke is associated with improved behavioral recovery, both in animal models^42^^,^^57^^,^^58^ and humans.^59^ Although hyperconnectivity has been demonstrated after both TBI^60^ and stroke,^61^ the functional relevance of this hyperconnectivity is unknown. We consider the functional implications of our connectivity findings below.

### 4.2 Temporal Dynamics of Remapped Circuits

Our results show an infarct size-dependent loss of synchronization, which we measure as neuronal fidelity, in areas of activation during stimulus-evoked responses. Larger strokes induce population-level neuronal fidelity decreases that progressively worsen from weeks 1 to 4, both in ipsilesional and contralesional cortices, whereas smaller strokes only show fidelity decreases ipsilesionally, as well as contralesionally during affected paw stimulation 1 week after stroke. It is likely that some of the changes in temporal dynamics and synchronization we observed are related to circuit remodeling, mentioned above, and damage to inhibitory networks. The activity of inhibitory interneurons and groups thereof are known to control network synchrony across the cortex, and damage to these gate-keeping networks, such as that caused by stroke, can lead to asynchrony in network dynamics across multiple timescales in humans.^62^^–^^64^ Moreover, increasing the order of polysynaptic connection of a circuit, which likely occurs during circuit remodeling that we have suggested, is involved in the case of larger strokes and adds increasing levels of noise,^62^^,^^65^ degrading signal strength and synchronization. Degradation of signal strength and synchronization likely compounds as the signal spreads across nodes of a network. Greater degrees of damage to these inhibitory networks probably produce greater disruptions in network synchronization, leading to more severe behavioral deficits.^64^

The decline in neuronal fidelity and synchronization in areas of activation during unaffected paw stimulation after larger strokes, which is absent in mice with smaller strokes, is even more perplexing. Areas of cortex that are activated during both unilateral affected and unaffected paw stimulation at week 4 after a larger stroke suggest that sensitivities or tuning of neuronal populations in these regions have begun to shift toward the affected paw. As neuronal tuning has been shown to impact firing rate,^66^^,^^67^ it is therefore plausible that firing rates of these cells are being affected by these tuning shifts. Furthermore, ischemia-inflicted damage to nodes, and specifically inhibitory interneuron networks, projecting to these contralesional regions may cause disinhibition within functionally related circuits, similar to that demonstrated in motor cortex strokes.^54^^–^^56^

### 4.3 Connectivity Changes after Stroke

In our analysis of RSFC changes after stroke, we observed substantial increases in the degree of connectivity of the unaffected $S1_{\mathrm{FP}}$ with much of the contralesional hemisphere after larger strokes, which is likely a spatial quantification of contralesional hyperexcitability.^68^^,^^69^ Activation topographies during affected and unaffected paw stimulation both mirror those of our resting-state functional connectivity maps. Similarly, Winship and Murphy^41^ demonstrated changes in neuronal selectivity to limb stimulation after focal stroke in the primary forelimb somatosensory cortex of mice, such that neurons that were previously selective to only contralateral hindlimb stimulation became more broadly tuned 1 month after stroke, processing sensory information from multiple limbs. These data show changes in functional activation over a period of weeks, similar to what we show here. Indeed, axonal sprouting and synaptogenesis have been shown to occur in the contralesional hemisphere from 3 to 14 days and from 14 to 60 days, respectively, after neocortical infarction in rats.^70^ These changes occurred contemporaneously with behavioral improvements, raising the possibility that neuroanatomical remodeling contributes to post-stroke recovery. Based on these data, we suggest that a combination of Hebbian-type plasticity and structural remodeling may be responsible for the significantly increased connectivity observed within the contralesional hemisphere.

The resting-state nature of our functional connectivity analyses does not provide any tuning or sensitivity information about these circuits. Most of the research concerning connectivity changes of stroke-affected circuits has focused on RSFC networks.^22^^,^^24^ Although changes in RSFC networks have been strongly correlated with functional recovery after stroke, the signal-to-noise ratio of resting-state activity (versus stimulus-evoked activity) can be low and potentially confounded by functionally irrelevant signals.^71^ Here, we also employ a different connectivity approach for prognosis: stimulus-driven bilateral coherence of functional circuits. We believe that the driving of functional circuits with peripheral sensory stimulation may render stimulus-driven bilateral coherence a more sensitive measure of functional network changes than RSFC. In addition, population-level neuronal calcium signals driven by peripheral stimulation have been shown to have greater fluorescence amplitudes than those of resting state activity.^72^ Our results show greater stimulus-driven bilateral coherence deficits in mice with larger strokes during affected paw stimulation, as well as significant deficits in stimulus-driven bilateral coherence during unaffected paw stimulation, an observation absent in mice with smaller strokes. These results further suggest an infarct size-dependent disruption of population-level neuronal synchronization caused by graded damage to inhibitory circuits,^63^^,^^64^ in addition to commenting on the importance of inhibitory networks within a functional domain in controlling spatially and temporally efficient global brain network function. Inhibitory neurons also play an important role in the propagation of slow waves across the cortex.^73^ In our experiments, bilateral coherence maps also show significant coherence of cortical areas involved in propagating slow waves of neuronal activity during affected paw stimulation at baseline that is significantly disrupted after stroke in mice, a finding that agrees with previous work.^32^ Our results build upon this, demonstrating an infarct size-based graded disruption of bilateral coherence during affected forepaw stimulation and a significant degradation of coherence during unaffected forepaw stimulation in mice with larger strokes. These data suggest that the efficiency of sensory-evoked wave propagation is dictated by the degree of spared functional cortical territory, and in turn inhibitory circuitry, that initiates the wave.

## 5 Conclusions

In summary, we have shown significantly different courses of repair and recovery after focal ischemic stroke of varying size in mice: a diffuse bilateral pattern of sensory-evoked remapping that predicts poor behavioral recovery and a focal ipsilesional activation pattern that predicts good behavioral recovery. These remapping patterns predict recovery independent of infarct size and may represent maladaptive versus adaptive plasticity. We also establish the importance of spatiotemporal dynamics of both affected and unaffected circuits, as well as brain networks including these circuits, in the prediction of functional recovery after stroke. These results inform and motivate new experiments investigating mechanisms that govern the repair of local circuits and global networks after stroke and how these mechanisms of repair contribute to functional recovery.

## Supplementary Material



## Data Availability

Data for this paper are stored on our institutional cloud storage platform and are available upon request, but access links must be generated upon request rather than persistent public links due to institutional policy on cybersecurity. The total data volume used for this paper is multiple terabytes in size. Please contact leejm@wustl.edu or aqbauer@wustl.edu for data requests.
