# Supplementary material for: Early changes in spatiotemporal dynamics of remapped circuits and global networks predict functional recovery after stroke in mice
Source: Neurophotonics. 2024 Dec 20;12(Suppl 1):S14604. doi: 10.1117/1.NPh.12.S1.S14604 (PMC11661640; doi:10.1117/1.NPh.12.S1.S14604)
Supplement: Supplementary file 1 [file NPh_012_S14604_SD001.pdf]

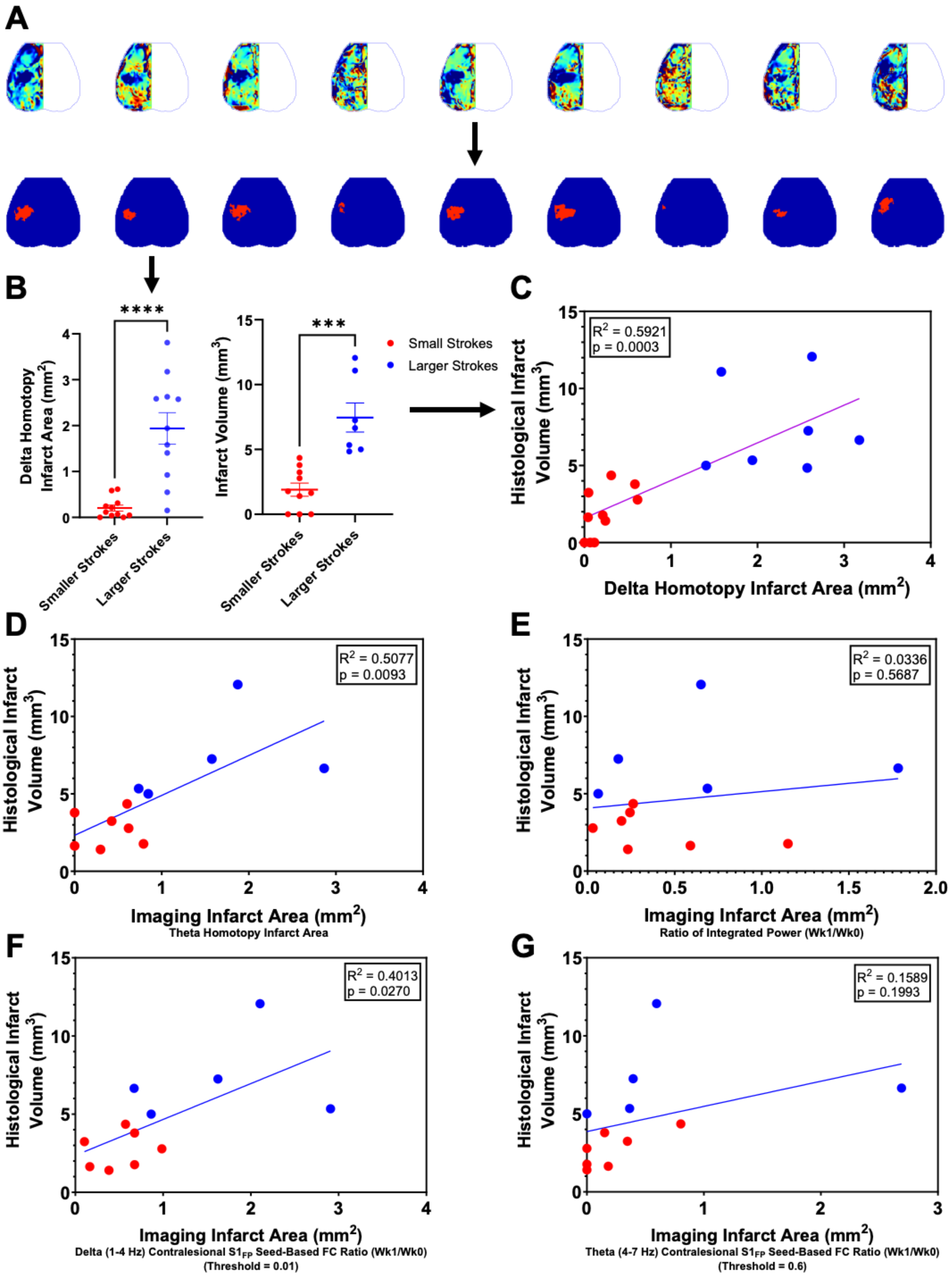
A
B
Delta Homotopy Infarct Area (mm2)
****
Smaller Strokes
Larger Strokes
Infarct Volume (mm3)
***
Small Strokes
Larger Strokes
C
R2 = 0.5921
p = 0.0003
Histological Infarct Volume (mm3)
Delta Homotopy Infarct Area (mm2)
D
R2 = 0.5077
p = 0.0093
Histological Infarct Volume (mm3)
Imaging Infarct Area (mm2)
Theta Homotopy Infarct Area
E
R2 = 0.0336
p = 0.5687
Histological Infarct Volume (mm3)
Imaging Infarct Area (mm2)
Ratio of Integrated Power (Wk1/Wk0)
F
R2 = 0.4013
p = 0.0270
Histological Infarct Volume (mm3)
Imaging Infarct Area (mm2)
Delta (1-4 Hz) Contralesional S1FP Seed-Based FC Ratio (Wk1/Wk0)
(Threshold = 0.01)
G
R2 = 0.1589
p = 0.1993
Histological Infarct Volume (mm3)
Imaging Infarct Area (mm2)
Theta (4-7 Hz) Contralesional S1FP Seed-Based FC Ratio (Wk1/Wk0)
(Threshold = 0.6)

Figure S1: Multiple Week 1 imaging measures predict histological infarct size at Week 8. (A), Individual delta homotopy infarct area maps (top) are shown for select mice in the larger stroke group, and binarized to obtain infarct ROIs (bottom). (B), Both delta homotopy infarct size ($p < 0.0001$) and histological infarct volume ($p = 0.0002$) of mice with larger strokes were significantly greater than the analogous measures for mice with smaller strokes. (C), Individual delta homotopy infarct areas are plotted against individual histological infarct volumes. (D), Individual theta (4-7 Hz) homotopy infarct areas are plotted against individual histological infarct volumes. (E), Individual areas of infarcts, obtained by applying a threshold to the pixel-wise ratio of integrated cortical GCaMP fluorescence power (0.01 – 7 Hz) from Week 0 to Week 1 at 0.2, are plotted against individual histological infarct volumes. (F), Individual infarct areas, obtained by applying a threshold to pixel-wise ratio of seed-based delta FC from Week 0 to Week 1 at 0.01, are plotted against individual histological infarct volumes. (G), Individual infarct areas, obtained by applying a threshold to the pixel-wise ratio of seed-based theta FC from Week 0 to Week 1 at 0.6, are plotted against individual histological infarct volumes. R_2 and p-values are displayed on each plot.

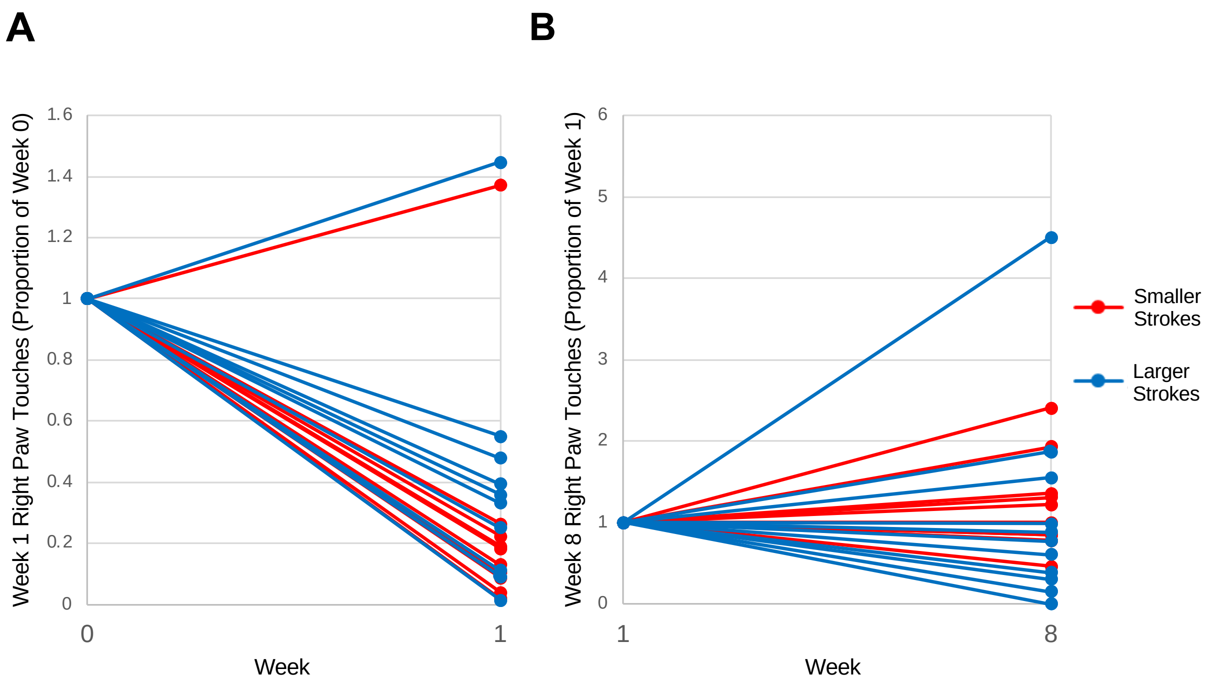

Figure S2: Individual Cylinder Rearing Touches. (A), The number of frames each mouse's right forepaw touched the cylinder during the cylinder rearing assay (relative to Week 0) is plotted at Week 0 and Week 1. Photothrombotic strokes occurred between Week 0 and Week 1 and demonstrates a general decline in right paw use. (B), The number of frames each mouse's right forepaw touched the cylinder during the cylinder rearing assay (relative to Week 1) is plotted at Week 1 and Week 8, and shows a range of recovery and decline in right paw use.

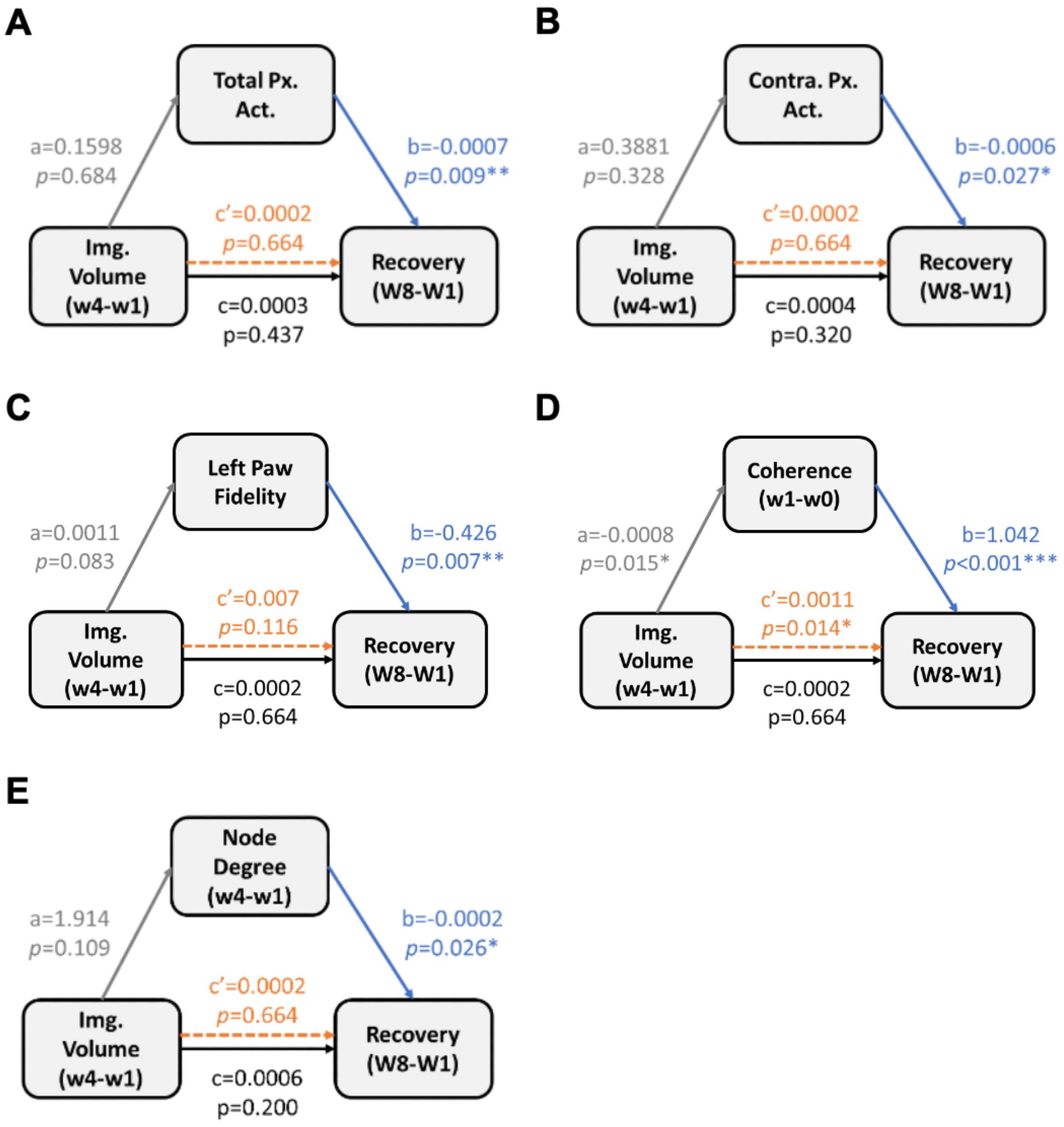

Figure S3: Early changes in dynamics of local circuits and global networks predict functional recovery independently of infarct size. (A-E), Tripartite diagrams of mediation analyses are shown illustrating the statistical effect of infarct size (as measured by delta homotopy infarct area) on functional recovery (as measured by changes in forepaw asymmetry in the cylinder rearing assay) mediated through the variables total pixel activation-area (A), contralesional pixel activation-area (B), unaffected paw fidelity changes (C), unaffected paw bilateral coherence changes (D), and Week 4 contralesional node degree (E). In each diagram, "a" represents the effect of infarct volume on the variable at the top of the diagram (termed the mediator), "b" represents the effect of the mediator on recovery (controlling for infarct size; the statistical effect of greatest interest), "c" represents the total effect of infarct size on recovery without controlling for the mediator, and "c' " represents the direct effect of infarct size on recovery, controlling for the mediator.

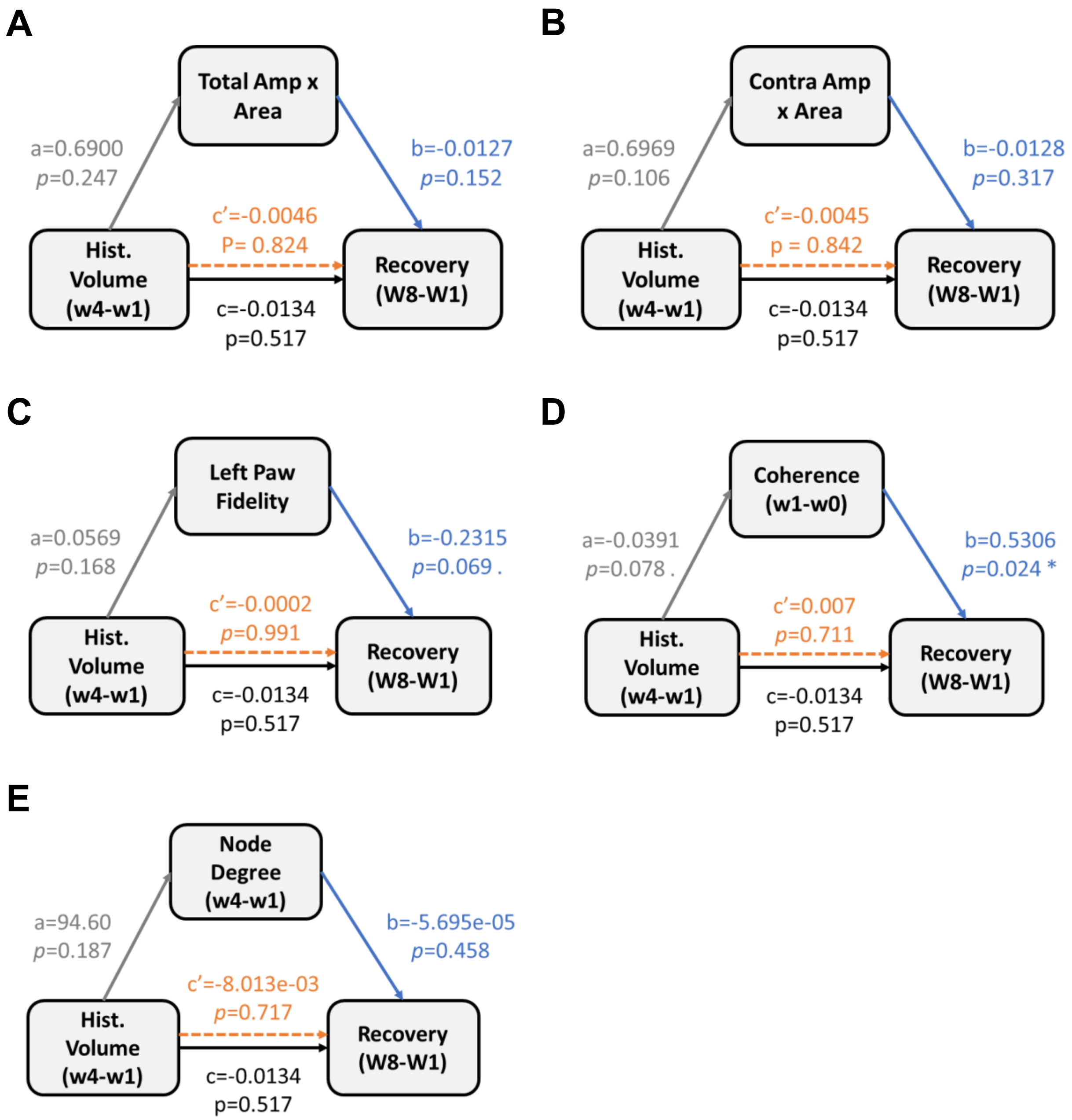

Figure S4: Using early changes in dynamics of local circuits and global networks to predict functional recovery with statistical correction for histological infarct volume. (A-E), Tripartite diagrams of mediation analyses are shown illustrating the statistical effect of histological infarct volume on functional recovery (as measured by changes in forepaw asymmetry in the cylinder rearing assay) mediated through the variables total pixel activation-area (A), contralesional pixel activation-area (B), unaffected paw fidelity changes (C), unaffected paw bilateral coherence changes (D), and Week 4 contralesional node degree (E). In each diagram, "a" represents the effect of infarct volume on the variable at the top of the diagram (termed the mediator), "b" represents the effect of the mediator on recovery (controlling for infarct volume; the statistical effect of greatest interest), "c" represents the total effect of infarct size on recovery

without controlling for the mediator, and “c’ ” represents the direct effect of infarct size on recovery, controlling for the mediator.
